# Sankara Nethralaya Diabetic Retinopathy Epidemiology and Molecular Genetic Study (SN--DREAMS III): *Study design and research methodology*

**DOI:** 10.1186/1471-2415-11-7

**Published:** 2011-03-24

**Authors:** Swakshyar Saumya Pal, Rajiv Raman, Suganeswari Ganesan, Chinmaya Sahu, Tarun Sharma

**Affiliations:** 1 Sankara Nethralaya, Vision Research Foundation, 18 College Road, Chennai-600006, Tamil Nadu, India

## Abstract

**Background:**

To describe the methodology of the Sankara Nethralaya Diabetic Retinopathy Epidemiology and Molecular Genetic Study III, an ongoing epidemiological study to estimate the prevalence of Diabetes and Diabetic Retinopathy in rural population of Kanchipuram and Thiravallur districts of Tamil Nadu, India and to elucidate the clinical, anthropometric, biochemical and genetic risk factors associated with diabetic retinopathy in this rural population.

**Methods:**

Sankara Nethralaya Diabetic Retinopathy Epidemiology and Molecular Genetic Study III will be a mobile van based epidemiological study; 11,760 participants aged ≥ 40 years will be recruited from the study areas. Eligible subjects will undergo blood sugar estimation to diagnose Diabetes. Oral Glucose Tolerance Test will be done to conform diabetes. All subjects with diabetes will undergo complete information of knowledge, aptitude and practice of diabetes and diabetic retinopathy, Diet questionnaire, demographic data, socioeconomic status, physical activity, anthropometric measurements, and risk of sleep apnoea. A detailed medical and ocular history, a comprehensive eye examination including refraction, slit lamp biomicroscopy examination, indirect ophthalmoscopy, slit lamp biomicroscopy, digital stereo fundus photography and ultrasound of eye will be done in the mobile van. Blood will be collected for biochemical investigations including blood hemoglobin, glycosylated hemoglobin, lipid profile, urea and creatinine, genetic study. Urine will be collected for microalbuminuria. All fundus photographs will be graded at base hospital. Participants who need treatment will be sent to the base hospital. A computerized database is created for the records.

**Conclusion:**

The study is expected to provide an estimate of the prevalence of Diabetes and Diabetic Retinopathy and also a better understanding of the genetic, anthropometric and socio-economic risk factors associated with Diabetic Retinopathy in a Rural South Indian population.

## Background

The number of adults with diabetes in the world is estimated to increase by 122%(135 million in 1995 to 300 in 2025).This increase is expected to be 42% in the developed world and 170% in the developing countries. India stands first with 195% (18 million in 1995 to 54 million in 2025)[1]. Diabetes mellitus was considered as an urban disease previously, however recent studies clearly shown increasing prevalence in rural areas as well [2-4]. Indian studies shows three fold increase in rural prevalence of diabetic in last one and half decade ( 2.2% in 1989 to 6.3% in 2003) [3]. In a cross sectional study of self reported population attending diabetic retinopathy screening camps it is found the rural prevalence of diabetes as 20% [4].

Diabetic retinopathy has been one of the foremost causes of blindness in the working age group of both developed and developing countries [1]. In India it is the 6^th ^cause of blindness at present which is increasing day by day. With increase of diabetics in India the prevalence of diabetic retinopathy also increasing [5].

Our previous report shows the prevalence of Diabetic retinopathy between rural and urban diabetic attending diabetic retinopathy screening camps which showed age adjusted prevalence of DR is 18% among both rural and urban diabetics [4].

Currently there are no population-based studies, which studied exclusively prevalence of diabetic retinopathy in rural area.

The present population-based study has been designed to answer the following questions:

1. What is the prevalence of diabetes mellitus in a rural south India population of, Tamil Nadu, India?

2. What is the prevalence of diabetic retinopathy in this well-defined population of diabetics, both newly diagnosed and previously diagnosed?

3. What are the risk factors--clinical, socio demographic, anthropomorphic, and biochemical--associated with diabetic retinopathy?

4. What sequence variants or polymorphisms in the RAGE, VEGF, PKC-*β*, and ApoE genes are associated with diabetic retinopathy?

This paper describes the study design and research methodology for SN-DREAMS-III

## Methods/Design

The study procedure is divided into five steps

Step I: Epidemiological field work

Step II: Training program, quality control, pilot study, definitions

Step III: Medical history questionnaire

Step IV: Ophthalmic examination, fundus photography, diabetic retinopathy classification

Step V: Biochemical investigations and genetic study

### Step I: Epidemiological Field work

This is an ongoing population-based cross-sectional study which commenced in June 2007, and data collection is likely to be completed by March 2010. The study has been approved by the Institutional Review Board of Sankara Nethralaya (Vision Research Foundation), Chennai, India.

#### a) Sample Size Estimation

Till now there are no true estimates of diabetic retinopathy among the rural population in India. Hence for the sample calculation we are using the best estimates that are available in literature. After analyzing various related studies, assuming a 2% prevalence of diabetic retinopathy among the rural population above the age of 40 yrs, the estimated sample size was 11760; keeping a design effect of 2 with precision of 80% and compliance of 80%.

#### b) Study Area

We have selected rural area of Kanchipuram and Thiravallur district of Tamilnadu, for conveniences. Each rural district has 8 talukas and 1,655 villages. We have randomly selected 26 villages, which are divided into 26 clusters, 13 from each of the districts. The definition of rural areas includes population of less than 5,000, population density of less than 400/square kilometre, and at least 25% of male population engaged in agricultural activities; a similar definition is adopted by the National Banks of India. The Demographic Data of the Study Areas is described in table 1

**Table 1:** Demographic Data of the Study Areas

Parameters	Kancheepuram		Thiruvallur	
		
	n	%	n	%
Population	2877468		2754756	
Male	1457242	**50.64**	1397407	**50.73**
Female	1420226	**49.36**	1357349	**49.27**
Literacy Rate	1952198	**67.84**	1867707	**67.80**
% Population ≥ 40 years	776871	**26.99**	716737	**26.02**
**Religion**				
Hindu	2583590	**89.79**	2476438	**89.90**
Muslim	113666	**3.95**	99408	**3.61**
Christian	170416	**5.92**	169719	**6.16**
Others	831	**0.03**	5713	**0.21**
Religion not stated	3679	**0.13**	3478	**0.13**

#### C) Sampling Method

A multi-stage cluster sampling method will be used in the study. A cluster will be defined as having a population of 1,200-2,000, and if the population of any village is found to exceed this number, the population is divided into 2 or more clusters.

#### d) Eligibility and exclusion criteria

People aged 40 years and above or those turning 40 in the current calendar year and resident at the target address for a minimum period of six months are included in the study. People staying at the target households for a period of less than six months, temporary residents (people who have permanent residence elsewhere), a resident who passes away after the enumeration but prior to examination, and an eligible resident who cannot be contacted after five physical attempts by the social worker are excluded from the study. Individuals who cannot be transported to the examination centre due to health reasons are excluded from the study.

#### e) Mapping and listing

A proper mapping and listing of the households is being done in a systematic manner to avoid omissions or duplications. A geographic map showing all major structures of the study area is being prepared, and all houses are numbered serially.

#### f) Household enumeration

Family members living on the same premises and sharing a common kitchen are defined as one household. A door-to-door survey of all the households on both sides of the street is conducted in the selected villages till we enumerate the calculated sample size. The household data sheet contains details of demography, educational qualification, occupation, residential status, and ocular and systemic disease, if any.

#### g) Appointments

Eligible subjects will be given an appointment date for an eye examination to be conducted in a mobile van, which is stationed at a convenient place. The mobile van has been designed to include all equipment that is necessary for the studies. All patients will be examined in the mobile van. The patients who need treatment are transported to the base hospital on an appointed date.

#### h) Refusals

A refusal will be recorded when the occupants of the household refuse to provide any information or refuse to comply with the examination even on repeated requests. A maximum of five attempts will be made to convince them to attend the study; if unsuccessful, they will be considered as dropouts.

#### i) Estimation of Fasting Blood Glucose

The team will visit the selected households a day in advance to request eligible individuals to observe a minimum of 8 hours of overnight fasting prior to estimation of the fasting capillary blood glucose levels the next morning. Using Accutrend Alpha, blood glucose estimation will be done on a sample of capillary blood obtained by finger-prick (Glucose Oxidase method). In accordance with the ADA criteria [6], subject with newly-detected diabetes is defined as the one having a fasting blood glucose ≥110 mg/dl. For patients who forget the fasting instructions and for dropouts, the fasting blood glucose will be estimated on Sundays or at their convenience.

### Step II: Training Program, Quality Control, Pilot Study, Definitions

#### a)Training Program

The epidemiology team of Sankara Nethralaya provided intensive training on a one-to-one basis to all the team members. This lasted for 7 days with 8 hours a day of training sessions. The aim was to ensure that each member of the team was well trained in doing a household survey, enumeration, and filling out the study data sheet. The trainees were instructed to use the sphygmomanometers and glucometer for estimating blood pressure and capillary blood glucose. The main objective was to avoid bias or errors in any of the procedures employed. Each trainee was evaluated individually and allowed to participate in the study only after he or she displayed minimum error rates for the tasks involved in the study.

#### b) Quality Control

In order to ensure accurate and reliable data, a comprehensive instruction manual has been prepared. A start-up training session helped us to standardize all the examination and diagnostic procedures. The glucometer is calibrated every day and its reproducibility is assessed by measuring the blood glucose for the same patient six times and also with two machines. A similar procedure is undertaken for the sphygmomanometer. The scale for measuring the weight is calibrated with a known weight once a week. The collected data is scrutinized manually before its entry into the computer.

#### c) Pilot Study

A pilot study was conducted taking 480 patients from the first village (Pyrarithangal); this was done to identify the initial practical difficulties in carrying out the study. The first step was the selection of the participants. Next, informed consent was obtained after the study had been explained to each participant and the interviewer was certain that the participant understood and accepted the contents. The subjects underwent the entire sequence of examination and laboratory procedures as planned for the actual study. During the pilot study, inter- and intraobserver variations for the photographic classification of diabetic retinopathy were compared (in order to minimize bias); kappa was 0.83 and 1.0, respectively. A Kappa value of > 0.8 suggests good agreement between the observers. A time-motion study was done to determine the time needed for a complete examination. The study revealed that it took 2 hours and 45 minutes for the collection of all the data, which included medical history, questionnaires and anthropometric measurements (45 minutes); ophthalmic work-up (1 hour); dilatation, fundus examination, and fundus photography (30 minutes); vibration perception threshold (VPT) testing and genetic work-up (30 minutes). The pilot study concluded that there was no questionnaire fatigue and a good response rate, creating the necessary confidence to proceed with the study.

#### d) Definitions

The various definitions taken in this study are summarised in the table 2

**Table 2:** Definitions

Provisional diabetics	New asymptomatic individual with a first fasting blood glucose level ≥110 mg/dl (Accutrend alpha)
	
Known diabetics	Diagonosis of diabetes made by a medical practitioner, or patient using hypoglycemic medication,either oral or insulin or both medication, either oral or insulin or both.
	
Newly diagnosed diabetics	Fasting blood glucose level ≥110 mg/dl on two separate days; provisional diabetics were retested at the base hospital by the laboratory method.
	
Duration of diabetes	Time interval between the date of diagnosis of diabetes (as made by a diabetologist or when the antidiabetic treatment started) and the date of eye examination.
	
Hypertension	If the systolic BP is ≥140 mm Hg or the diastolic BP is ≥ 90 mm Hg or the patient is on antihypertensive treatment.
	

### Steps III: History and Examination procedures

#### a) Medical history and questioner

A detailed questionnaire will be administered regarding the medical history, a general physical examination, habitual history and educational and occupation history. The data in the medical history include duration and treatment of diabetes or hypertension, a family history of diabetes, coronary artery disease, symptoms related to diabetic nephropathy or neuropathy, the presence of an ear lobe crease, and duration of total sleep. The ocular history includes details of the first and last eye examination, present or past ocular complaints, and laser treatment or ocular surgery. Tobacco and alcohol history includes duration, type, amount, and age at start, present and past status. Occupation and education history also taken.

#### b) Ophthalmic examination

##### I) Visual Acuity and Refraction

The modified ETDRS chart (Light House Low Vision Products, New York, NY,USA) is used to test the distance visual acuity; and for those who cannot read the English alphabet, Landolt's ring test is used. If the visual acuity is less than 4/4 (logMAR 0.0), the pinhole visual acuity is assessed. Objective refraction is performed with a streak retinoscope (Beta 200, Heine, Germany) followed by subjective refraction. If the subject is unable to read the 4/40 (logMAR 1.0) line, vision is checked at one meter. If he or she is still unable to identify any of the largest optotypes, perception of hand movements is observed. If hand movements cannot be identified, the examiner checks for perception of light, which is recorded as present or absent.

##### II) External Examination

External examination is performed using a handheld flashlight. The face and eyes are examined for the presence of strabismus, extra ocular movement abnormalities or any other gross abnormality.

##### III) Slit-Lamp Examination

The Topcon SL-D7 ( Topcon corporation, Japan) slitlamp is used. Using a moderately wide beam, the eyelids, margins, lashes, canthi, and puncta are systematically examined, followed by the palpebral and bulbar conjunctiva, sclera and cornea. Then, using a narrow parallelopiped beam, the cornea, anterior chamber and iris are examined for abnormality. Grading of peripheral anterior chamber depth is done according to the Van Herick grading.The iris and pupillary margins are examined under high magnification to look for iris neovascularization.

##### IV) Applanation Tonometry

Using the Goldmann applanation tonometer (Hagg-streit AT 900, Hagg-streit AG, Switzerland ), the intraocular pressure (IOP) is measured in both eyes; 0.5% proparacaine eyedrops are used for topical anesthesia, and a 2% fluorescein strip to stain the tear film [7].

##### V) Gonioscopy

Gonioscopy is performed in dim ambient illumination with a shortened slit that does not fall on the pupil. A Sussmann-type 4-mirror handheld gonioscope (Volk Optical Inc, Mentor,Ohio,USA) is used. Gonioscopy is performed only on those patients who have an elevated intraocular pressure, iris neovascularization or a shallow anterior chamber (Van Herrick's grading ≤ 2). The angle is graded according to the Shaffer grading; the peripheral iris contours, degree of trabecular meshwork pigmentation and angle neovascularization are also recorded. In eyes with an occludable angle, laser iridotomy is performed before dilatation; the rest of examination is done at a later date [8].

##### VI) Grading of Lens Opacities

The subject's pupils are dilated with 5% phenylephrine and 1% tropicamide eyedrops; if phenylephrine is contraindicated, 1% cyclopentolate eyedrops are used. Grading of lens opacities is performed using the Lens Opacities Classification System60 (LOCS chart III, Leo T. Chylack, Harvard Medical School, Boston, MA). Lenticular opacities are graded by comparison with the standard set of photographs, which are retroilluminated by mounting on a light box. With the slit beam at a 45◦ angle, the slit height and width are adjusted to approximate the overall brightness of the corneal image and anterior subcapsular zone to nuclear color/opalescence standard N1; 0◦ brightness is allowed to visualize all the lens opacities without causing discomfort to the patient. Keeping the slit width at 0.2 mm, nuclear opalescence (NO) and nuclear color (NC) are graded by comparing the slit-lamp examination image with the nuclear standards NO1 to NO6 and NC1 to NC6. Cortical cataract (C) and posterior subcapsular cataract (P) are graded by examining the opacity in retroillumination images (0◦ angle) focused either anteriorly (at the iris plane) or posteriorly (at the plane of the posterior capsule) and comparing these examination pictures to the cortical standards C1 to C5 and posterior subcapsular standards P1 to P5.

##### VII) Fundus Examination and Clinical Grading

The binocular indirect ophthalmoscope (Keeler Instruments Inc., Pennsylvania, U.S.A) and +20 D lens (Nikon) are used to examine the fundus. The macular area is specifically examined with a +78 D lens (Nikon) to document any pathology.

##### VIII) Fundus Photography

Considering fundus photography is the gold standard, to document and diagnose diabetic retinopathy, all individual will undergo 45 degree 4 field photograph. The Zeiss, Visucam, fundus camera with visupac digital image archiving system will be used to photograph fundus of subjects. Atleast 6 mm pupil dilatation ( with tropicamide 0.5% and phenylephrine 5%) will be insured before fundus photography. No image manipulation will be done before or during grading. Images will be stored as uncompressed jpeg files, copied to CDs and will be sent for grading. Independent photo grading of digital fundus photography by retinal specialist will be done.

##### Fundus Photography Using Stereo-Pictures

Irrespective of the presence or absence of diabetic retinopathy, 45◦ four-field stereoscopic digital photographs (posterior pole, nasal field, superior and inferior) are taken for all subjects with a Carl Zeiss fundus camera (Visucamlite, Jena, Germany). However, for those who are found to have diabetic retinopathy on clinical examination, additional 30◦ seven-field stereodigital pairs are taken.

##### IX) Evaluation of Photographic Grading and Quality

The photographic grading and quality will assessed using the Visupac digital image archiving system. The "Screenscope" (Berezin Stereo Photography Products, Mission Viejo, CA, USA), a stereo viewer that can be fixed on a computer monitor, is used to examine the stereo pairs. Photographs of each eye are reviewed and given grades for overall quality. Field definition and image clarity are graded as inadequate for reading or grading, adequate (sufficient visualization of disc, macula, and vessels to grade), and good (small vessels or retinal details visible across 90% of the image) [9].

##### x) Photographic Grading of Diabetic Retinopathy

The modified classification of diabetic retinopathy based on the degrees of retinopathy used by Klein et al [10] (Table 3) is used; digital photographs are assessed and graded by three independent observers (experienced retinal specialists) in a masked fashion.

**Table 3:** Classification of diabetic retinopathy

Level	Definition
1	No retinopathy
1.5	Retinal hemorrhages only, no microaneurysms
2	Micro aneurysms (1 or more) only
3	Micro aneurysms and 1 or more of the following: retinal hemorrhages, but total of hemorrhages and microaneurysms (H/MA) less than standard (STD) photograph 2A, hard exudates (HE) less than STD photograph 3, soft exudates (SE) questionably present; intraretinal microvascular (IRMA) abnormalities questionably present; venous beading (VB) questionably present; or small venous loops definitely present
4	Micro aneurysms and 1 or more of the following but definition of level 5 not met: H/Ma greater than or equal to STD photograph 2A, HE greater than or equal to STD photograph 3, SE definitely present, IRMA definitely present, VB definitely present; large venous loops or reduplication definitely present^--^
5	IRMA definitely present in four peripheral fields and greater than or equal to STD photograph 8A in 2 or more fields; or, again in all 4 peripheral fields any three of the following: H/MA greater than or equal to STD photograph 2A in 1 field, SE definitely present in 2 or more fields, IRMA definitely present in 2 or more fields, or VB definitely present in 2 or more fields.
6	Fibrous proliferation or new vessels on or within 1 disc diameter (DD) of the disc graded less than photograph 10A, new vessels else where of any extent or pre retinal or vitreous hemorrhage, but level 7 definition not met
7	Diabetic Retinopathy Study (DRS) high-risk characteristics include one or more of the following: new vessels elsewhere greater than one-half disc area in any single photographic field and pre retinal hemorrhage or vitreous hemorrhage in any field; new vessels on or within 1 DD of the disc graded less than photograph 10A with pre retinal or vitreous hemorrhage; new vessels on or within 1 DD of the disc graded greater than or equal to photograph 10A with or without pre retinal or vitreous hemorrhage.

##### Step V: Biochemical Investigations

In case of patients with provisional diabetes, confirmation will be done by re-estimation of the fasting blood glucose by enzymatic assay Biochemical analysis is done on the Merck Micro Lab 120 semi-automated analyzer. Total serum cholesterol (CHOD-POD method), high-density lipoproteins (CHOD-POD method after protein precipitation), serum triglycerides (CHOD-POD), hemoglobin (calorimetric hemoglobinometer), packed cell volume (capillary method) and the lycosylated hemoglobin fraction (Bio-Rad DiaSTAT HbA1c Reagent Kit) will be estimated. Microalbuminuria estimation will be done by a semi-quantitative procedure (Clintek 50 Bayer Urine Analyzer) with the first morning urine sample.

#### Genetic studies

**Figure 1:**
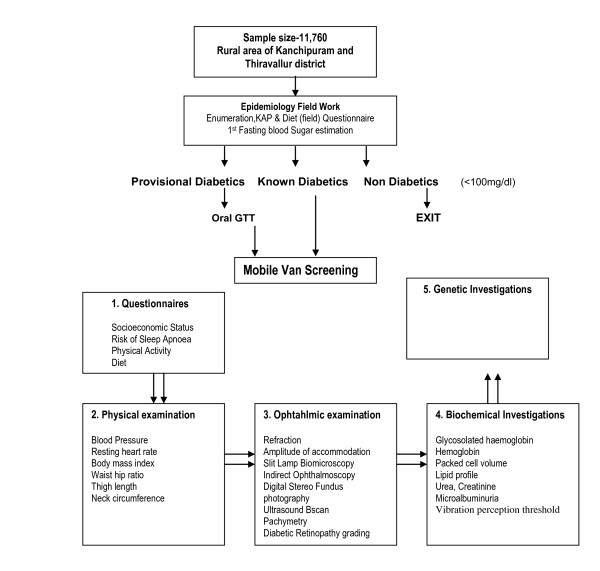
**Pattern of study organization**.

Genome-wide association studies are a promising new tool for deciphering the genetics of complex diseases. SN-DREAMS III would identify the possible candidate genes through a whole genome association study in the south Indian population. Subgroup of type 2 diabetic patients of ≥ 10 years duration with sight threatening diabetic retinopathy will be taken as case and type 2 diabetic patients of ≥ 15 years duration, without diabetic retinopathy will be taken as control. After obtaining their informed consent, genomic DNA will be isolated. Genotypes for each study subjects will be done on the Affymetrix platform. The outcome will generate data that would allow the understanding of the various genetic factors that are possibly involved in the disease pathogenesis. An overview of the methodology is provided in figure. 1.

#### Data management

The data entered by the social workers, optometrists, ophthalmologist, and lab technician will be counter checked for any deficiencies by their respective colleagues. The person responsible for the checking the details will have to sign at the end of the page of each questionnaire. Before the subject leaves the examination centre, the entire questionnaire will be checked for any deficiencies. The data from the questionnaire will be entered in the Microsoft Access. The data willl be doubly entered with limit checks to avoid wrong entries

Data analysis will be conducted using standard statistical techniques for comparing two independent groups: chi-squared tests for equality of proportions, independent t-test for equality of means, Wilcoxon rank sum test, multiple logistic and linear regression, and proportional hazards modeling. The distribution of continuous variables will be assessed by measures of normality and graphical displays so that non-parametric methods or data transformations may be applied when appropriate.

### Special features of SN-DREAMS III

1) It will be a population based epidemiological study, so accuracy of data will be much well defined 2) we have taken a well defined definition of rural population 3)photographic grading for all subject, which is gold standard. In presence of significant cataract, cataract surgery followed by fundus photography is being done.

## List of Abbreviations

SN-DREAMS: Sankara Nethralaya Diabetic Retinopathy Epidemiology and Molecular Genetic Study, DR: Diabetic Retinopathy, RAGE: Receptor for Advanced Glycation End products, VEGF: Vascular Endothelial Growth Factor, PKC: Protein Kinase C, ApoE: Apolipoprotein E, ADA: American Diabetes Association, VPT: Vibration Perception Threshold, ETDRS: Early Treatment Diabetic Retinopathy Study, LogMAR: Logarithm of Minimum Angle of Resolution, IOP: Intra Ocular Pressure, LOCS: Lens Opacities Classification System, NO: Nuclear Opalescence, NC: Nuclear Cataract, C: Cortical Cataract, P: Posterior subcapsular cataract, CHOD-POD: Cholesterol Oxidase- Peroxidase, DNA: Deoxyribo Nucleic Acid.

## Competing interests

The authors declare that they have no competing interests.

## Authors' contributions

Dr SSP, Dr GSI, Dr CAS were involved in data collection, Dr RRN was in charge of data maintenance, Dr TS was the overall supervisor of the project. All authors have read and approved the final manuscript.

## Pre-publication history

The pre-publication history for this paper can be accessed here:

http://www.biomedcentral.com/1471-2415/11/7/prepub

## References

[B1] King H Aubert RE Herman WH Global burden of diabetes, 1995-2025 prevalence, numerical Estimates and projection Diabetes Care 1998 21 1414 31 10.2337/diacare.21.9.1414 9727886

[B2] Ramachandran A Jali MV Mohan V Snehalatha C Viswanathan M High prevalence of diabetes in an urban population in South India BMJ 1988 297 587 90 10.1136/bmj.297.6648.587 3139221 PMC1834545

[B3] Ramachandran A Snehalatha C Dharmaraj D Viswanathan M Prevalence of glucose intolerance in Asian Indians: Urban-rural difference and significance of upper body adiposity Diabetes Care 1992 15 1348 55 10.2337/diacare.15.10.1348 1425100

[B4] Rani PK Raman R Sharma V Mahuli SV Tarigopala A Sudhir RR Analysis of a comprehensive diabetic retinopathy screening model for rural and urban diabetics in developing countries Br J Ophthalmol 2007 91 1425 9 10.1136/bjo.2007.120659 17947265 PMC2095459

[B5] Dandona L Dandona R John RK Estimation of blindness in India from 2000 through 2020: implications for the blindness control policy Natl Med J India 2001 14 6 327 34 11804362

[B6] Diagnostic criteria for diabetes mellitus Diabetes Care 2003 26 Suppl 1 5S

[B7] Kass MA Standardizing the measurement of intraocular pressure for clinical research--Guidelines from the Eye Care Technology Forum Ophthalmology 1996 103 183 185 8628552 10.1016/s0161-6420(96)30741-0

[B8] Kanski JJ The Glaucomas Kanski JJ Clinical Ophthalmology--A Systematic Approach 1999 4 Oxford, Boston:Butterworth-Heinemann 183 262

[B9] Early Treatment of Diabetic Retinopathy Study Report No.1 Photocoagulation for diabetic macular edema Arch Ophthalmol1985 103 796 806

[B10] Klein R Klein BEK Magli YL An alternative method of gradingdiabetic retinopathy Ophthalmology 1986 93 1183 1187 3101021 10.1016/s0161-6420(86)33606-6

